# Past and future epidemic potential of chikungunya virus in Australia

**DOI:** 10.1371/journal.pntd.0009963

**Published:** 2021-11-16

**Authors:** Timothy White, Gina Mincham, Brian L. Montgomery, Cassie C. Jansen, Xiaodong Huang, Craig R. Williams, Robert L. P. Flower, Helen M. Faddy, Francesca D. Frentiu, Elvina Viennet

**Affiliations:** 1 Centre for Immunology and Infection Control, School of Biomedical Sciences, Queensland University of Technology, Kelvin Grove, Queensland, Australia; 2 Research and Development, Australian Red Cross Lifeblood, Kelvin Grove, Queensland, Australia; 3 Research and Innovation Services, University of South Australia, Adelaide, South Australia, Australia; 4 Metro South Public Health Unit, Metro South Hospital and Health Service, Brisbane, Queensland, Australia; 5 Communicable Diseases Branch, Queensland Department of Health, Herston, Queensland, Australia; 6 UniSA Clinical & Health Sciences, University of South Australia, Adelaide, South Australia, Australia; 7 School of Health and Behavioural Sciences, University of the Sunshine Coast, Petrie, Queensland, Australia; University of California, Davis, UNITED STATES

## Abstract

**Background:**

Australia is theoretically at risk of epidemic chikungunya virus (CHIKV) activity as the principal vectors are present on the mainland *Aedes aegypti*) and some islands of the Torres Strait (*Ae*. *aegypti* and *Ae*. *albopictus*). Both vectors are highly invasive and adapted to urban environments with a capacity to expand their distributions into south-east Queensland and other states in Australia. We sought to estimate the epidemic potential of CHIKV, which is not currently endemic in Australia, by considering exclusively transmission by the established vector in Australia, *Ae*. *aegypti*, due to the historical relevance and anthropophilic nature of the vector.

**Methodology/Principal findings:**

We estimated the historical (1995–2019) epidemic potential of CHIKV in eleven Australian locations, including the Torres Strait, using a basic reproduction number equation. We found that the main urban centres of Northern Australia could sustain an epidemic of CHIKV. We then estimated future trends in epidemic potential for the main centres for the years 2020 to 2029. We also conducted uncertainty and sensitivity analyses on the variables comprising the basic reproduction number and found high sensitivity to mosquito population size, human population size, impact of vector control and human infectious period.

**Conclusions/Significance:**

By estimating the epidemic potential for CHIKV transmission on mainland Australia and the Torres Strait, we identified key areas of focus for controlling vector populations and reducing human exposure. As the epidemic potential of the virus is estimated to rise towards 2029, a greater focus on control and prevention measures should be implemented in at-risk locations.

## Author summary

Chikungunya virus (CHIKV) is transmitted primarily by *Aedes aegypti* and *Aedes albopictus* mosquitoes and causes a potentially debilitating febrile and arthralgic disease. The virus is a threat to public health in regions where the primary vectors are established, as evidenced by past epidemics in the Indian Ocean Islands, South America and the Caribbean. In Australia, there are established populations of *Ae*. *aegypti* both on the mainland and in the Torres Strait, and of *Ae*. *albopictus* in the Torres Strait. This provides a theoretical potential for CHIKV transmission, as seen historically with dengue virus (DENV). It is therefore important to understand the epidemic potential of CHIKV in Australia. We estimated the basic reproduction number (*R*_*0*_) of CHIKV during the years 1995–2019 for 11 Urban Centres and Localities (UCLs) in Australia, and found that Brisbane, Cairns, Darwin, Rockhampton, Thursday Island, and Townsville were all susceptible to CHIKV epidemics. We then forecasted epidemic potential from 2020–2029 and found an increase in *R*_*0*_ across the six main UCLs. By highlighting factors that significantly influence the epidemic potential of CHIKV in Australia, our study supports evidence-based decision making for vector control and public health programs.

## Introduction

Chikungunya is an often-debilitating disease caused by chikungunya virus (CHIKV), a mosquito-borne alphavirus of the *Togaviridae* family. The Makonde word “chikungunya” means “that which bends up”, denoting the symptom of arthralgia common in many cases. Other symptoms include fever, rash and nausea, with rarer cases of chronic joint pain [1–3]. Infection with CHIKV is self-limiting in immunologically competent individuals, however chronic morbidity is often observed [2,4]. CHIKV is transmitted by several mosquito species, particularly two highly invasive species of the *Aedes* genus (Subgenus: *Stegomyia*) *Aedes aegypti* and *Aedes albopictus*. Urban cycles of transmission involve these species because they live in close proximity to human habitation and possess biological and ecological attributes that increase their vectorial capacity [5,6]. Epidemic CHIKV activity is linked to urban cycles of mosquito-human transmission, often in locations previously naïve to the virus. Both mosquito species are present in some regions of Australia and are capable of expanding their geographical distributions, providing the opportunity for CHIKV outbreaks into novel Australian regions [7].

Since it was first identified in Tanzania in 1952, CHIKV has expanded in distribution with numerous outbreaks recorded across Africa, Asia, the Americas, and islands of the Indian Ocean [8]. CHIKV phylogenetically comprises into three main genotypes: West-African, Asian and East-Central-South-African (ECSA), along with a more recently identified sub-lineage of ECSA, the Indian Ocean Lineage (IOL). A 2005–06 outbreak in La Réunion, with the primary vector being *Ae*. *albopictus*, resulted in an estimated 244,000 confirmed cases with an attack rate of 35% and mortality rate of 0.03% [9]. The identification of a point mutation (E1-A226 A to V) in the ECSA genotype was hypothesised to shorten the extrinsic incubation period (EIP) of the IOL and result in rapid transmission by *Ae*. *albopictus* [10]. An outbreak in Italy in 2017 resulted in an average incidence rate of 0.0068% for the region, but up to 0.335% in towns such as Anzio [11]. Outbreaks of the Asian genotype in the Caribbean islands in 2013–15 resulted in an attack rate of 0.6% and a mortality rate of 0.024% in hospitalized cases (with no vector species stipulated) [12]. The severity of these outbreaks highlights the potential risk for other locations with suitable conditions for transmission.

CHIKV can be introduced into a new region by a viraemic traveller or importation of infected mosquitoes. After an initial outbreak is contained or becomes self-limited, CHIKV activity may subside due to large-scale immunity in the human host population, greatly reducing the epidemic potential [6]. CHIKV transmission is influenced by climatic factors, such as temperature and rainfall, along with human-vector interactions. Currently there are no licensed vaccines for CHIKV, so immunologically naïve populations that have suitable conditions and vectors are at risk. Treatments are also limited to targeting symptoms of fever and overall joint pain [4]. Predicting outbreaks and measuring the risk of CHIKV transmission in different locations are of public health importance to prioritize appropriate surveillance and preventative measures.

The Asia-Pacific region has a high incidence of CHIKV activity [3], posing a risk to neighbouring regions where climate conditions are suitable, and an abundant and competent vector population is present. In the Pacific region, vulnerable locations include the Torres Strait Islands with established populations of *Ae*. *albopictus* and *Ae*. *aegypti* [13] and parts of Queensland (Australia), with established *Ae*. *aegypti* [14–16], with expansion through national and international freight pathways. These regions do not currently have endemic CHIKV transmission but imported cases of Asian lineage and IOL CHIKV have been increasing in recent years, consistent with increased virus activity in the Asia-Pacific [17, 18]. The full extent of CHIKV epidemic potential in Australia has not previously been modelled but is theoretically possible in many locations.

Historically, Australian populations of *Ae*. *aegypti* [14] have vectored endemic dengue virus (DENV) up to the 1940s [19]. The implementation of vector control has facilitated the elimination of this vector outside Queensland [20] and from the region of south-east Queensland [21]. *Aedes aegypti* has high potential to facilitate CHIKV transmission in Australia, due to the anthropophilic nature of the vector [22]. However, the epidemic potential of CHIKV in various regions in Australia is largely unknown. Additionally, the replacement of wild-type *Ae*. *aegypti* populations in Cairns, Townsville, and surrounding towns with *Wolbachia*-infected *Ae*. *aegypti* may decrease transmission risk. However, the epidemic potential in Australia would potentially be broader if vector species distributions expand are re-configured by mainland invasion by *Ae*. *albopictus*, or *Wolbachia* infection of *Ae*. *aegypti* populations in north Queensland regions is lost.

Although no documented cases of transfusion-transmitted CHIKV have been reported [23], CHIKV remains a theoretical threat to blood supply safety. Understanding the epidemic potential of local CHIKV transmission will allow better and timely management of threats to blood safety and assessment of public health risk. Our aims were to i) evaluate the past potential of CHIKV transmission in *Ae*. *aegypti* in specific locations of Australia, by estimating basic reproduction number (*R*_*0*_) for each location; ii) forecast potential transmission in these locations for 2020–2029; and iii) estimate and understand how environmental factors and virus genotype can impact transmission by *Ae*. *aegypti*. This will identify variables with the greatest impact on CHIKV epidemic risk, adding to the evidence base necessary for effective vector control measures and epidemic response programs (e.g. Queensland Chikungunya Management Plan [24]).

## Methods

### Study definition

**Fig 1:**
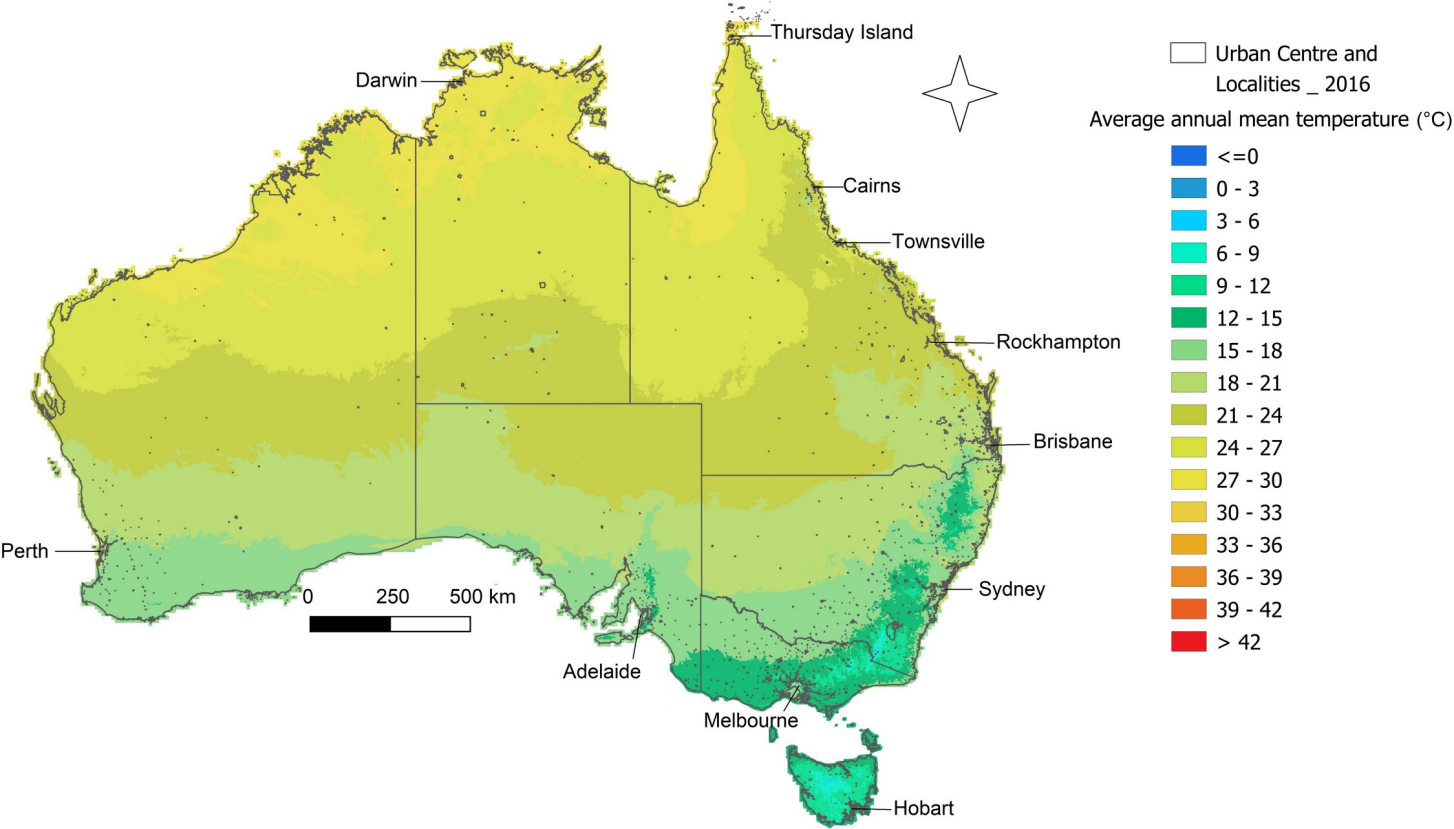
Map of Australia and Torres Strait identifying the UCLs of focus. Study locations are based on the Australian Bureau of Statistics UCL land definitions. The data used to create this figure has been sourced from the Bureau of Meteorology.

To evaluate CHIKV epidemic potential within Australia from 1995–2019, we analysed epidemiological and environmental parameters. Eleven locations, here referred to as urban centres and localities (UCLs), across Australia (Fig 1) were chosen based on current and past *Ae*. *aegypti* presence or absence (determined by Australian public health authorities using trap-based mosquito surveillance at these locations). These locations were Cairns, Rockhampton, Thursday Island and Townsville which have confirmed *Ae*. *aegypti* populations; Adelaide, Hobart and Melbourne which have no recorded populations; and Brisbane, Darwin, Perth and Sydney which have historically recorded populations but now no longer do.

### Data collection

Data on temperature and human population for each UCL were gathered from publicly accessible Australian government websites, whilst values for vector biting and transmission rates, along with infectious and extrinsic incubation periods were determined from published research (Table 1). To accurately predict the potential for transmission, the Asian genotype (hereafter named AL) and IOL are considered here separately, as they are the most likely to be introduced to Australia due to geographic proximity [17].

**Table 1:** Equation variable descriptions with corresponding values/equations used.

ID	Description	Equation/Values			Reference
***b***	Vector bite rate (average per day)	0.0043*T+0.0943			[34]
***ß***_***m***_	Human-to-vector transmission rate (per day)	AL		0.98	[35]
		IOL		0.92	[15]
***ß***_***h***_	Vector-to-human transmission rate (per day)	AL		0.56	[35,36]
		IOL		0.64	[15]
***M***_***L***_	Mosquito population density (females seeking bloodmeal per hectare)	Estimated using the CIMSiM model			CIMSiM
***H***_***L***_	Human population density (per hectare)	Calculated from linear regression of census years from ABS			[26] (ABS)
***γ***	Human infectious period (days^-1^)	AL	Worst-case limit	7	[37]
			Best-case limit	2	
		IOL	Worst-case limit	5	[38]
			Best-case limit	2	
***μ***	Vector mortality rate (deaths per day)	0.8692−0.159*T+0.01116*T^2^−0.0003408*T^3^+0.000003809*T^4^			[34]
***τ***	Extrinsic incubation period (days)	AL	Best-case limit	11.36	[39]
			Worst-case limit	2.75	
		IOL	Best-case limit	11.63	
			Worst-case limit	3.39	
***c***	Vector control efficiency (rate of mosquito survival after control methods)	Worst-case limit		0.3	[40,41]
		Best-case limit		0.1	
***T***	Mean Temperature	From Australian BOM recordings			[25]

AL = ‘Asian Genotype of CHIKV’, IOL = ‘Indian Ocean Lineage of CHIKV’, CIMSiM = ‘Container-Inhabiting Mosquito Simulation’, ABS = ‘Australian Bureau of Statistics’, BOM = ‘Bureau of Meteorology’

The monthly average maximum and minimum temperatures for the 11 UCLs were obtained from the Australian Bureau of Meteorology (BOM), from January 1995 until September 2019 (S1 Fig) [25].

Human population density was calculated using population data and UCL land area size collected from the Australian Bureau of Statistics (ABS) for the census years 1996, 2001, 2006, 2011 and 2016 [26]. Linear regression was performed using Microsoft Excel to estimate data for non-census years starting at 1995 through to 2019. *Ae*. *aegypti* population density was predicted using the container-inhabiting mosquito simulation (CIMSiM) software [27], which utilises minimum and maximum temperature values [25], rainfall and humidity data [25], along with census data for human population [26], access to water-bearing containers (used as mosquito breeding sites) and access to nutritional requirements. The simulation then returns host-seeking female *Ae*. *aegypti* population data that were used for this study. The data on water-bearing containers and nutritional inputs were developed as part of earlier calibration studies for the CIMSiM model in the Australian context [28,29]. In this work, a representative breeding container profile was established and mosquito productivity for these containers was field validated for use in Australia. Thus, these model settings in CIMSiM were maintained for all simulation locations.

The remaining values required for the modelling component: vector biting rate, vector mortality rate, human-to-vector transmission rate, vector-to-human transmission, infectious period, extrinsic incubation period, and vector control rate, were retrieved from research and literature review sources (Table 1). To be as accurate as possible, sources were selected if they investigated AL or IOL CHIKV for *Ae*. *aegypti*, under conditions that closely matched experiments from other research groups, for the viral parameters human-to-vector and vector-to-human transmission rates, infectious period, and extrinsic incubation period. The vector-to-human transmission rate parameter had multiple sources contributing to the data, so values were selected based on relevance (use of *Ae*. *aegypti* and AL or IOL CHIKV strains), then averaged to provide the most accurate prediction of possible outcomes. For research conducted using *Ae*. *aegypti* populations in other regions of the world, results were selected based on the strains most similar to those in Queensland. For example, parameter estimates from experiments conducted on Caribbean species were selected due to evidence of genetic similarity with Asian *Ae*. *aegypti* [30].

### Estimating historical potential for CHIKV

The *R*_*0*_ was estimated and used as the predictor of CHIKV transmission potential in humans. *R*_*0*_ predicts the number of subsequent infections from one infection in a susceptible human population, so that a *R*_*0*_ < 1 means transmission cannot be maintained, where *R*_*0*_ > 1 means transmission is maintained during an outbreak. *R*_*0*_ = 1 identifies endemic transmission. An equation to estimate Zika virus epidemic potential, proposed by Villela *et al*. [31] was adapted for Zika virus (ZIKV) and DENV epidemic potential in Australia [32]. We used the same adapted equation for estimation of CHIKV. As both CHIKV genotypes have slight variances in values, a different equation for *R*_*0*_ and each genotype was developed, written here as *R*_*AL*_ (Eq 1) and *R*_*IOL*_ (Eq 2).


$$R_{AL}=\frac{b^{2}\times {ß}_{mAL}\times {ß}_{hAL}}{\gamma \times \mu (1+\tau_{AL}\times \mu )}\times \frac{M_{L}}{H_{L}}\times c$$ (1)

$$R_{IOL}=\frac{b^{2}\times {ß}_{mIOL}\times {ß}_{hIOL}}{\gamma \times \mu (1+\tau_{IOL}\times \mu )}\times \frac{M_{L}}{H_{L}}\times c$$ (2)

As well as investigating two genotypes of CHIKV, the upper and lower limits of the parameters human infectious period, extrinsic incubation period and vector control efficiency were considered, so that best- and worst-case scenarios of transmission in *Ae*. *aegypti* were predicted (Table 1). The best-case scenario was calculated by using the limits of the variables that decrease transmission rate, whilst the worst-case scenario was calculated using the limits of the variables that increased transmission rate (S1 Table). With the two outer limit scenarios identified, all other scenarios within those limits were excluded from the results. The standard deviation was also calculated for the best- and worst-case scenarios to represent monthly variation. Collectively, the genotype of CHIKV, and the best- and worst-case limits, combined with the temperature variation of each UCL, were expected to accurately predict CHIKV potential transmission by *Ae*. *aegypti* in Australia. Here, the *R*_*0*_ was applied as a tool for assessing relative risk between UCLs and scenarios, with the theoretical value of 1 providing a reference line for transmission sustainability.

The “R Software” (version 3.6.1) [33] was used to estimate the *R*_*0*_ for each CHIKV genotype, then to forecast this value from 2020 to 2029. The remaining values and equations (with associated upper and lower limits) were also assigned to their respective variables. We estimated best- and worst- case scenarios for each UCL, where best-case related to the limits of the variable values that resulted in lower transmission and worst-case related to the values that resulted in higher transmission. These two scenarios were employed for analysis as they comprehensively outlined the limits of CHIKV potential in Australia. Our predictive model assumes that each locality had a population of *Ae*. *aegypti* and that this was the only vector population present, that the human population was immunologically naïve to CHIKV, and that the mosquito population was not infected with *Wolbachia* or possessed any other trait that may reduce transmission potential.

### Sensitivity analysis of variables

To quantify the relationship and impact of each variable on the *R*_*0*_ equation, we performed uncertainty analysis to analyse variance, coupled with sensitivity analysis (SA). For uncertainty analysis, we explored Monte Carlo (MC) methods and produced a matrix of 100,000 sample values within the respective ranges for each of the test variables through Latin Hypercube Sampling (LHS) [42]. Then, an additional column of 100,000 *R*_*0*_ values were calculated based on these sample values, creating the uncertainty ranges for the *R*_*0*_ values. Sensitivity analysis was then performed on the samples (S2 Table) using the Partial Rank Correlation Coefficients (PRCC) method to analyse the density and variance of each variable, along with the impact of each on the final *R*_*0*_ value [42]. As an MC method of uncertainty was used for simulation, a global SA method can be used to efficiently evaluate the variation in each variable, instead of a local SA method which only considers values close to the average range. The PRCCs were determined between the *R*_*0*_ value and all variables, as well as between variables.

### Forecast model construction

To forecast future *R*_*0*_ values from 2020–2029, we utilised the same methodology as the historical data set. We gathered the predicted variation to temperature (min and max) and total rainfall for each month of the ten-year period [43], and predicted relative humidity changes for each season (three month period) [44] for the 11 UCLs. As the predicted variations are calculated relative to the reference period of 1986–2005, the datasets for this period were collected for each variable [45]. Then the predicted change in variable was applied to the reference data, allowing us to estimate the future values for each variable. The predicted human population data was calculated in the same way as the historical dataset; using a linear regression from census years to predict values for every year from 2020–2029. At this point, the entomological population for each UCL was estimated using the CIMSiM and adult host-seeking female population was used for the *R*_*0*_ equation. The final predicted values required for the *R*_*0*_ equation were the vector bite rate and vector mortality rate, which were both calculated using the mean predicted temperature for each UCL. The same *R*_*0*_ equation was utilised for the forecast data (2020–2029), which resulted in a best- and worst-case scenario for each UCL and genotype of CHIKV.

## Results

### Historical *R*_*0*_

**Fig 2:**
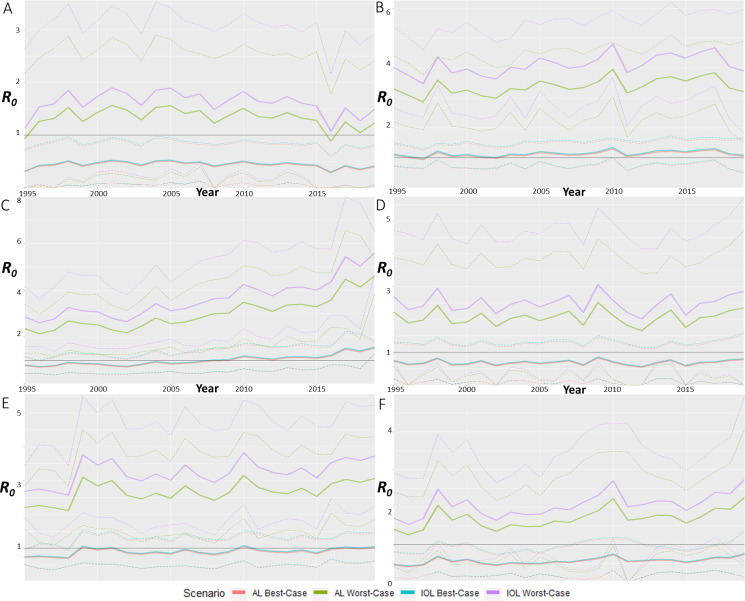
*R*_*0*_ estimations for best- and worst-case scenarios from 1995 to 2019 for the significant UCLs. CHIKV *R*_*0*_ from 1995 to 2019 by averaging monthly estimations for each year for A) Brisbane, B) Cairns, C) Darwin, D) Rockhampton, E) Thursday Island and F) Townsville. Best- and worst-case scenarios were estimated using the limits of each variable from the *R*_*0*_ equation, where best-case correlates with least transmission and worst-case correlates with most transmission of CHIKV. Each scenario also has upper and lower standard deviation limits, with averages in bolder lines.

We estimated that only six of the 11 UCLs (Brisbane, Cairns, Darwin, Rockhampton, Thursday Island, Townsville) recorded relevant *R*_*0*_ sufficient for analysis. The remaining five UCLs (Adelaide, Hobart, Melbourne, Perth, and Sydney) were estimated to have *R*_*0*_ of, or closely equal to, zero. These UCLs with *R*_*0*_ values sufficient for analysis have confirmed presence of *Ae*. *aegypti*, apart from Brisbane and Darwin which have historical presence of the vector. Although still an interesting finding, to allow for easier visualisation of the higher *R*_*0*_, we removed the five UCLs with *R*_*0*_ <1 from graphical representation. Of the six remaining UCLs, each had higher *R*_*0*_ values for IOL compared to AL, despite sharing almost identical trend patterns between strains and scenarios (Fig 2).

Brisbane was the only UCL with a *R*_*0*_ for the best-case scenario for both CHIKV strains below 1. The average trendline fluctuated within a fixed range, with *R*_*0*_ fluctuating between 1 and 2 for worst-case scenarios for both strains, whilst in the best-case scenarios for both strains *R*_*0*_ fluctuated between 0.3 and 0.5 (Fig 2A). The predicted scenarios here suggested that Brisbane could have sustained transmission of either strain only if environmental conditions were favourable, with 1995 and 2016 being the years where transmission was least favourable.

Cairns was the only UCL where the best-case scenario average was almost consistently above *R*_*0*_ of 1, however the lower limit of the SD remained below *R*_*0*_ of 1 (Fig 2B). The Cairns worst-case scenario displayed one of the highest *R*_*0*_ values predicted throughout all UCLs and remained consistently high, around 4 and 3 for IOL and AL, respectively. The predicted scenarios for Cairns suggested that this UCL was the most likely to sustain CHIKV transmission across any scenario, where only the months of May through September were unsuitable for transmission under best-case scenarios.

Darwin was the other UCL with the highest *R*_*0*_ values predicted for worst-case scenarios, however the pattern showed a *R*_*0*_ that increased over time by 2.5 units (Fig 2C). The upper SD bound of this trend reached *R*_*0*_ values of around 8, making it the UCL with highest *R*_*0*_ prediction. The upward trend showed a shift for the best-case scenarios from *R*_*0*_ values below 1 through to 1.5. The *R*_*0*_ potential for Darwin increased over time, to the point where 2019 would have had a *R*_*0*_ greater than 1 for every scenario.

The trend for Rockhampton displayed a fluctuation pattern around *R*_*0*_ of 2 and 3 for worst-case scenarios and just below 1 for best-case (Fig 2D). The lower SD limits for best- and worst-case scenarios reached *R*_*0*_ of 0 or lower (but these are considered equal to zero) in 1996, 2002, 2011, 2012, 2015 and 2019. This indicated that while Rockhampton had a consistent potential for CHIKV activity, transmission was unsustainable in the months of August and September, regardless of the scenario.

For Thursday Island, we estimated a fluctuating trend after an increase in epidemic potential before the year 2000 (Fig 2E). For the worst-case scenario, the *R*_*0*_ ranged from 2.5 to 3.5, with SD upper limits around 5 and lower limits around 1.5, indicating large inter-yearly variation in potential. The best-case scenario trend fluctuated around 1 after the initial increase, with upper SD limits around *R*_*0*_ of 1.5 and 0.5 for lower SD limits, also suggesting that the month of the year was important for transmission potential.

The potential for transmission in Townsville fluctuated while also displaying an upward trend over time (Fig 2F). The SD limits of the worst-case scenario suggested large monthly variation of potential, with the months of June, July, August, and September not supporting transmission (*R*_*0*_ less than 1). The best-case scenario average fluctuated around *R*_*0*_ of 0.5 with only the upper SD limit rarely reaching *R*_*0*_ > 1. The potential for transmission was dependent on the scenario, but also the monthly climate.

### Sensitivity analysis of variable uncertainty

**Fig 3:**
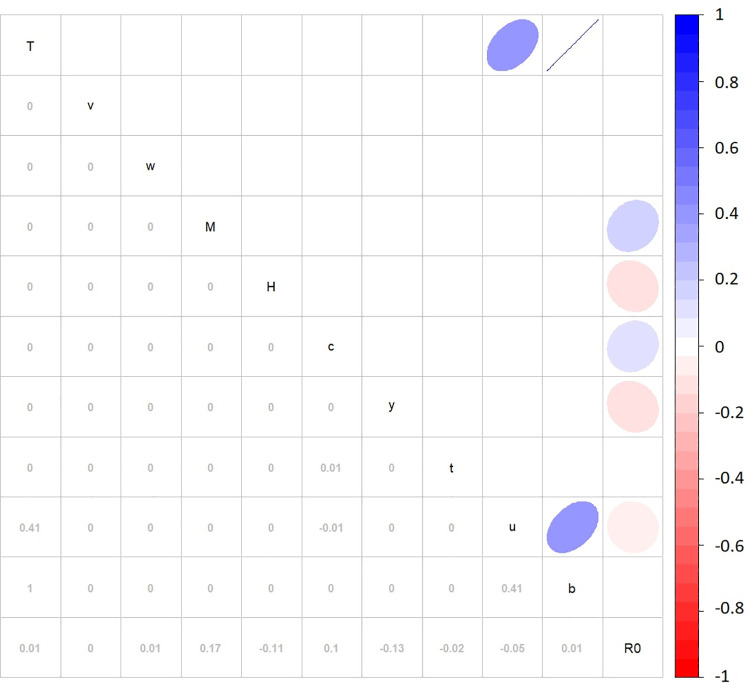
Sensitivity analysis using correlation matrix of *R*_*0*_ variables. Sensitivity analysis was performed by Partial Rank Correlation Coefficient using Monte Carlo methods and Latin Hypercube Sampling. Numeric and graphic displays of the correlation coefficient are displayed in the lower left-hand side segment and the upper right-hand side, respectively. The colour and direction of ellipse relates to the degree of correlation between parameters. The variables are displayed diagonally, where *T* is average temperature, *v* is human-to-vector transmission rate, *w* is vector-to-human transmission rate, *M* is mosquito population density, *H* is human population density, *c* is vector control efficiency, *y* is infectious period, *t* is extrinsic incubation period, *u* is vector mortality rate, *b* is vector biting rate, and *R*_*0*_ is the basic reproduction number.

The sensitivity analysis of the variables (Fig 3) highlighted significant low positive correlations on *R*_*0*_ values for mosquito population density (*M*) and vector control efficiency (*c*) (R^2^ = 0.17 and 0.1 respectively), along with low negative correlations for human population density (*H*), infectious period (*y*), vector mortality rate (*u*) and EIP (*t*) (R^2^ = -0.11, -0.13, -0.05 and -0.02 respectively). Average temperature (*AvgT*) had higher significant positive correlations on vector mortality rate (*u*) and vector biting rate (*b*) (R^2^ = 0.41 and 1.0 respectively). The positive correlation between vector biting rate and vector mortality rate (R^2^ = 0.41) identified collinearity, but not a causation relationship.

### Forecast model

**Fig 4:**
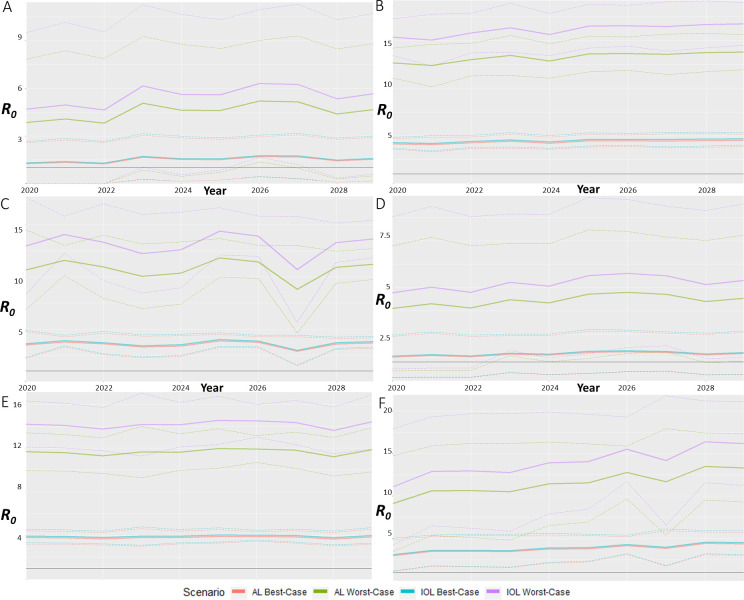
*R*_*0*_ forecast estimations for each significant UCL. Forecasts of the best- and worst-case scenarios of *R*_*0*_ for A) Brisbane, B) Cairns, C) Darwin, D) Rockhampton, E) Thursday Island, and F) Townsville for 2020 to 2029. Forecasts are calculated using the *R*_*0*_ equation with bold lines representing the yearly average and dashed lines representing the monthly variation as standard deviation.

To forecast the future epidemic potential for 2020 to 2029, we utilised the *R*_*0*_ equation to estimate the theoretical transmission potential for each UCL. Once again, the forecasts showed that IOL scenarios had higher *R*_*0*_ values than AL scenarios. Brisbane was forecasted to have stable trends, with each scenario having a varying average *R*_*0*_ of 1 (Fig 4A). The monthly variation displayed as standard deviation showed a difference in *R*_*0*_ up to 5 for worst-case scenarios. The average best-case scenarios for Brisbane were both above *R*_*0*_ of 1. Cairns also displayed stable trends, with fluctuation of *R*_*0*_ of 1 for scenario averages (Fig 4B). All scenario averages and standard deviations were above *R*_*0*_ of 1, with worst-case scenarios among the highest average forecasted at *R*_*0*_ of 17. Greater variation in average scenario *R*_*0*_ was forecasted for Darwin, with variation up to 3 for worst-case scenarios (Fig 4C). Despite such variation the average *R*_*0*_ still forecasted a level trend across the time-period. Again, all scenarios were above *R*_*0*_ of 1, with the highest values around *R*_*0*_ of 14. Rockhampton was forecasted to also be a level trend for the average scenario values, with fluctuation in *R*_*0*_ of 1 (Fig 4D). The highest value was around *R*_*0*_ of 6, with monthly variation for every scenario passing below *R*_*0*_ of 1. Thursday Island displayed level trend lines for each scenario, with high values around 14.5 for worst-case scenarios (Fig 4E). The best-case scenarios were stable around *R*_*0*_ of 4. The monthly variation for Thursday Island was around *R*_*0*_ of 2 for worst-case scenarios and 0.5 for best-case. Townsville was the only UCL to forecast an upward trend in *R*_*0*_ values, with an increase in *R*_*0*_ of 5 for worst-case scenarios (Fig 4F). The monthly variation for worst-case scenarios was around *R*_*0*_ of 7, but despite such large variation, all values remained above the *R*_*0*_ of 1 threshold.

## Discussion

The emergence of CHIKV over recent decades has seen numerous epidemics of varying severity across tropical and temperate regions where mosquito vector populations are established. Although Australia has not experienced CHIKV outbreaks, it is important to understand how risk of CHIKV transmission varies in the different regions, particularly over shorter future timeframes, to enable effective public health management and mitigation. Our study suggests that the six most northern UCLs investigated (Brisbane, Cairns, Darwin, Rockhampton, Thursday Island and Townsville) had varying historical and future potential for CHIKV epidemics facilitated by *Ae*. *aegypti*. The historical potential varied amongst UCLs and across best- and worst-case scenarios but remained consistent with IOL CHIKV having higher *R*_*0*_ values compared to AL CHIKV. We showed that sustained epidemics in Brisbane, Rockhampton, Thursday Island and Townsville were scenario dependent, meaning that worst-case scenarios were likely to sustain epidemics whereas best-case scenarios were not likely to sustain epidemics. Cairns and Darwin had *R*_*0*_ higher than 1 for most of the best-case scenarios, highlighting them as the most likely to sustain epidemics under all scenarios, with only colder months (May-September) not supportive. Our findings are in congruence with the historical distribution of *Ae*. *aegypti* in Australia. Cairns, Rockhampton, Thursday Island and Townsville have confirmed mosquito populations, while Brisbane and Darwin, have historically recorded populations which demonstrate the potential of CHIKV transmission if suitable conditions were to be met. Adelaide, Hobart, and Melbourne have no recorded populations of *Ae*. *aegypti* which also concord with our results. Finally, while Perth, and Sydney have historically recorded *Ae*. *aegypti* populations, the vector is no longer established in either of these UCLs. Overall, these observations are consistent with the susceptibility to DENV transmission in the past reported for Brisbane, Cairns, Darwin, Rockhampton, Thursday Island and Townsville [19].

Sensitivity analysis highlighted that mosquito population density, human population density, vector control efficiency and infectious period had a significant impact on the *R*_*0*_ estimates. The forecasting of CHIKV outbreaks for each UCL highlighted that the northern-most UCLs, Brisbane, Cairns, Darwin, Rockhampton, Thursday Island, and Townsville, had the highest *R*_*0*_ forecasts. Brisbane and Rockhampton were forecasted to have the lowest *R*_*0*_ values of these UCLs, which supports the relationship between lower mean maximum temperatures and rainfall in colder months, and reduced potential for CHIKV epidemics. Cairns and Thursday Island were forecasted to have consistent *R*_*0*_ values regardless of seasonality, while Brisbane, Darwin, Rockhampton, and Townsville were forecasted to have seasonal variation of *R*_*0*_. Townsville was the UCL forecasted to have the highest *R*_*0*_ values, making it among the most likely to sustain an outbreak relative to other UCLs investigated. As Australia is in the Southern hemisphere, the Northern UCLs are subject to, on average, hotter climates. This supports the findings of this study, where UCLs with average hotter temperatures were identified to have higher *R*_*0*_ values, both in historical and future estimations.

Temperature is an important driver of arbovirus transmission, but rainfall, proximity to human dwellings, and availability of container habitats are also significant for vector populations [5,14,21,46]. Here we show that *Ae*. *aegypti* vector population density has a positive correlation on *R*_*0*_. Additionally, the other temperature-dependent variables, namely vector mortality rate and vector biting rate, have correlations with *R*_*0*_ also. We show that UCLs that have average daily temperatures around 27–31°C and high rainfall (leading to increased vector population density) have higher *R*_*0*_ values. However, mosquito behaviours are known to be temperature-dependent, with negative correlations above 35°C for blood feeding and flight [47], and when host and environmental temperatures are similar [48]. These temperature-dependant behaviours were relevant to the overall prediction validity of Cairns, Darwin, and Thursday Island, as these UCLs often enter temperature ranges that may adversely impact mosquito fitness in summer months. Water storage containers in Brisbane are predicted to provide suitable environments for overwintering of *Ae*. *aegypti* [49] which supports survivability despite the negative correlations of lower temperatures on the vector.

Thursday Island (and other inhabited islands of the Torres Strait) has populations of *Ae*. *albopictus*, a species that is more efficient at transmitting CHIKV-IOL. As we assumed that the strains will be transmitted only by *Ae*. *aegypti*, the prediction for Thursday Island does not account for transmission by *Ae*. *albopictus* and, therefore we may have underestimated the epidemic potential of IOL. Conversely, some regions of north Queensland, including Townsville and Cairns, now possess established populations of *Wolbachia*-infected mosquitoes [50,51] along with comprehensive and continued mosquito control programs, which may reduce transmission likelihood. We excluded this from the vector control parameter as the nature of this measure means that arbovirus transmission is predicted to be low while the *Wolbachia*-infected *Ae*. *aegypti* population remains established [51–53]. Additionally, the potential contribution of CHIKV outbreaks caused by involvement of *Ae*. *albopictus* that may play a role in the future is not considered in our forecasts. Although this mosquito is frequently detected at shipping ports in Australia, it has thus failed to establish on the mainland. Through sensitivity analysis we identify the key drivers of the *R*_*0*_ to be the vector and human populations densities, along with the vector control efficiency and the infectious period. Not all significant variables driving transmission can be managed, but by controlling vector populations and human exposure the *R*_*0*_ can be reduced.

Our study has some limitations. The definition of some of UCLs by the Australian Bureau of Statistics changed throughout the census years, altering estimates of the resulting human population density. The impact of this on our estimates of final *R*_*0*_ is unlikely to have been large as the variation in UCL boundaries was small. The reliance on CIMSiM is validated, but only proven accurate for predicting populations of northern Queensland (where *Ae*. *aegypti* is present). Therefore, we assumed that it is accurate for other UCLs as *Ae*. *aegypti* survivability is supported [49] more southerly than previously predicted by CIMSiM. Factors relevant to Australia that we were unable to take into account include the variation in available larval container habitats (e.g. deteriorating rainwater tanks) over time, the frequency of incursion of vectors in Australia, the warming climate, and other entomological factors (e.g. ability for *Ae*. *aegypti* to overwinter). Last, the timing of the study meant that data for 2019 were only analysed until September, resulting in the final three months being excluded from the calculation of averages for this year.

The historical absence of CHIKV in Australia creates complexity in validating the risk model utilised in this study, however, the partial presence of DENV allows for some comparison. Based on the similarities of DENV and CHIKV, we theorise that transmission patterns would be similar in respective UCLs based on climate suitability. Moreover, this study is an extension of the study published by Watson-Brown *et al*. (2019) in which this same model has been used and validated with DENV and ZIKV [32]. Our findings correlate also here with historical presence of DENV in Australia, providing partial validation of our model. The additional inclusion of findings from ZIKV transmission estimation in Australia [54] also identified similar UCLs of importance when assessing risk. The presence of the shared vector, *Ae*. *aegypti*, in Australia allows for the accurate comparison and validation of these arboviruses. The regression of DENV transmission to Northern Queensland, along with continued containment of CHIKV and ZIKV at entry points to Australia, reinforces the efforts of policy makers. These efforts have ensured that Australia has not had endemic or epidemic CHIKV, despite transmission potential of CHIKV in certain regions being clearly identified by our work here. We suggest that the focus of management and resources be focused on susceptible UCLs foremost, supported by past DENV transmission and ZIKV estimations of similar nature [54].

Here we have selected localities to predict theoretical potential of CHIKV epidemics in Australia. These Australian estimates are novel as there are no existing predictions for CHIKV. We have shown that epidemics of two major lineages of CHIKV were theoretically possible in Australia. From 1995 to 2019, epidemics were theoretically possible in Cairns, Townsville, and Rockhampton. CHIKV transmission was also theoretically possible in Darwin and Brisbane if *Ae*. *aegypti* were to re-establish in these regions and supports a strategic emphasis on surveillance programs in regions vulnerable to invasion to detect incursions early to attempt eradication [20,55,56]. *Aedes aegypti* is also a primary vector for other arboviruses including DENV and ZIKV and therefore this study supports comparisons of their respective transmission potentials. ZIKV epidemic potential has previously been estimated in Australia using similar parameters and methodologies, with the same six UCLs found to have high historical epidemic potential. An analysis of the threat of ZIKV to blood supply safety identified that donors from susceptible UCLs contribute only a small percentage of blood donor supply [54]. Taken together, the results from our study and the previous ZIKV work suggest that these UCLs are of greatest concern for sustaining transmission of multiple arboviruses [32]. There are no documented cases of transfusion-transmitted CHIKV [23]. However, CHIKV transmission through blood transfusion is theoretically possible. Therefore, this virus could pose a threat directly to blood safety, and indirectly on donor attendance. Our CHIKV predictions and forecasts confirm and support the need for continued importation security measures for infective humans and mosquitoes, along with vector control programs in UCLs with established vector mosquitoes and contribute to future blood safety management policies. The framework employed in this study can also be adopted by other countries/locations with established vector populations but no current CHIKV epidemic activity, to analyse risk and make evidence-based decisions for prioritizing regional vector surveillance and suppression programs.

## Supporting information

S1 Table Parameter values for each scenario and the corresponding outcome *R*_*0*_ equation values. Parameter values are Year, Month and UCL specific to create scenarios shown. AL = ‘Asian Genotype’, EIP = ‘Extrinsic Incubation Period’, IOL = ‘Indian Ocean Lineage’, LB = ‘Lower Bounds’, R0 = ‘Basic Reproduction Number’, UB = ‘Upper Bounds’.
(CSV)

S2 Table Statistics for each parameter in sensitivity analysis. (CSV)

S1 Fig Monthly mean maximum and minimum temperatures for each UCL for the study period 1995–2019. A) Adelaide, B) Brisbane, C) Cairns, D) Darwin, E) Hobart, F) Melbourne, G) Perth, H) Rockhampton, I) Sydney, J) Thursday Island, K) Townsville.
(TIF)
