# Supplementary figures and images for: Past and future epidemic potential of chikungunya virus in Australia
Source: PLoS Negl Trop Dis. 2021 Nov 16;15(11):e0009963. doi: 10.1371/journal.pntd.0009963 (PMC8631637; doi:10.1371/journal.pntd.0009963)

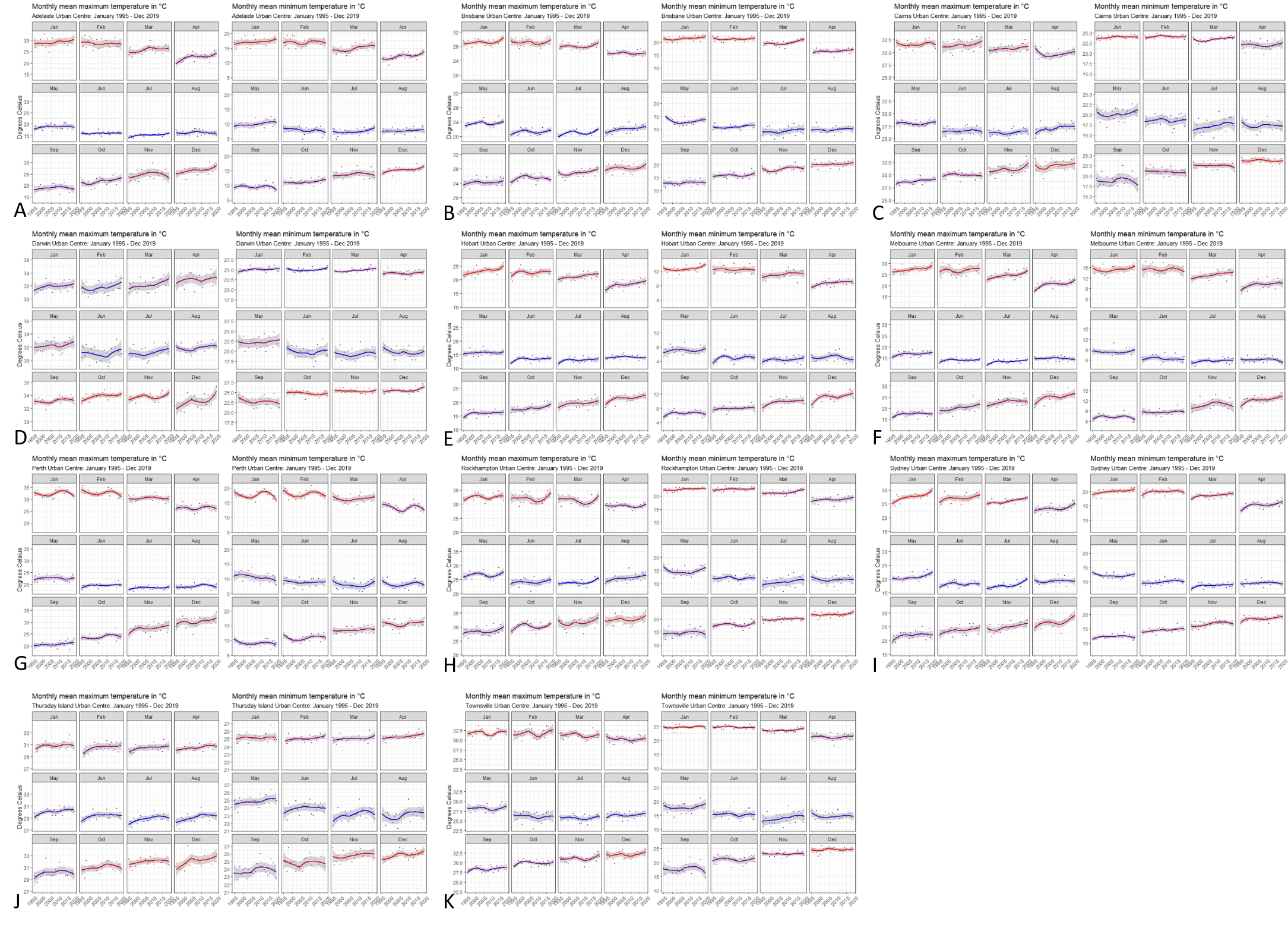

Supplement: S1 Fig — A) Adelaide, B) Brisbane, C) Cairns, D) Darwin, E) Hobart, F) Melbourne, G) Perth, H) Rockhampton, I) Sydney, J) Thursday Island, K) Townsville. (TIF) [file pntd.0009963.s003.tif]
